# Hakka culture brand image design based on the human–computer interaction model

**DOI:** 10.3389/fpsyg.2022.956615

**Published:** 2022-08-25

**Authors:** Rui Chen

**Affiliations:** College of Arts and Design, Gannan Normal University, Ganzhou, China

**Keywords:** brand image design, human–computer interaction model, Gannan Hakka culture, visual elements, Hakka culture

## Abstract

In order to better disseminate Hakka culture, this thesis focuses on the cultural dissection of Hakka culture in Gannan and the development and reflection of Hakka culture in Gannan. This article aims to design the brand image of Hakka culture through human–computer interaction model. And based on this, this article discusses the brand concept and the elements of cultural and creative products and discusses the connection between Hakka cultural and creative and tourism brands. At the same time, this article conducts an in-depth study on the creative design of the Hakka cultural tourism brand in Gannan. First, this article analyzes the brand connotation and analyzes the connotation and constituent elements of brand cultural and creative products. Second, this article analyzes the relationship between Hakka culture and tourism brand cultural and creative product design. It discusses how to inherit and develop Hakka culture from the perspective of how Hakka culture is integrated into brand cultural and creative products. Third, this article analyzes the design practice of cultural and creative products of the Hakka cultural tourism brand in Gannan. This article first analyzes the visual image design of the cultural and creative products of the Hakka cultural tourism brand in Gannan. Then it analyzes the design of a series of cultural and creative products of the Hakka cultural tourism brand in Gannan. The experimental results show that the popularity of different products is more than 50%, which shows the effectiveness of this design.

## Introduction

Hakka culture is a unique culture of the Han people, and its tourism development value is huge. Gannan has very rich and unique Hakka cultural tourism resources. Cultural tourism expression is a difficult point in the development of cultural tourism resources, and tourism cultural and creative products can achieve the purpose of boosting local economic development and are also an important carrier of cultural dissemination. In recent years, the design of Hakka cultural tourism cultural and creative products has been greatly improved, but there are still many products with outdated packaging, inadequate product image design, and similar packaging design. Therefore, through in-depth understanding of the current consumer demand, it further integrates the representative elements of Hakka culture into the design process of cultural and creative products for creative ideas. It is, indeed, necessary to make cultural and creative products more vivid and interesting and to reflect the cultural connotation. The purpose of this research is to combine the design of cultural and creative products of tourism brands with Hakka culture. This article explores the innovative design of the visual image of Gannan Hakka tourism brand cultural and creative products, and the use of tourism communication channels. It enhances the tourism value, cultural value, and popularity of the Hakka culture in Gannan. According to the extracted visual symbols of Hakka culture in Gannan, it combines Hakka culture in Gannan with cultural and creative products of tourism brands. It innovates the design of Gannan Hakka cultural and creative products, expands the influence and dissemination of Gannan Hakka culture in the tourism market, and enhances its emotional memory among tourism consumers. Since the Hakka culture in Gannan is the inheritance and development of the Chinese and Han cultures, its purpose is to promote the traditional Chinese and Chinese culture while spreading the Gannan Hakka culture.

Research on brand image design has been ongoing, and Hong conducted a survey on consumers’ satisfaction with the spatial expression in the glasses flagship store. He proposed a brand experience space design scheme that can improve consumer satisfaction and brand awareness (Hong and Han, 2017). Gryshchenko (2020) provided methodological tools for forming the brand capital of innovative universities. Lee studies the scalability of user brand experience through color analysis of over-the-counter drug packaging (Lee et al., 2017a). Nedergaard (2018) aimed to explain the structure of design management practice that relies on the implementation of brand innovation strategies in collaboration with external design consultants. Such research results are not enough to meet people’s direct interaction with brands, so they need to be realized through human–computer interaction models.

Next, some studies related to human–computer interaction models will be introduced. Bascetta and Ferretti (2019) introduced a newly conceived method to design admittance filters for manual control tasks to ensure the stability of human–computer interaction. Truong and Yoshitaka (2018) proposed a novel structured recurrent neural network (S-RNN) to simulate the spatiotemporal relationship between human subjects and objects in everyday human interactions (Truong and Yoshitaka, 2018). Chen (2017) analyzed and modeled the interface interaction of the self-service terminal based on distributed cognition theory. He determined the relationship between interaction and information presentation in human–computer interaction and proposed a self-service terminal interface interaction design method. In order to solve the problem of enhancing human factors by human–computer interaction in sports APP, Hu (2017) proposed a fusion method of multivariate fuzzy implication. The above research is only at the theoretical level and has not been fully practiced. Therefore, this article combines the two modules of human–computer interaction model and brand image design to study the Hakka culture brand image design based on human–computer interaction model.

This research is to verify the feasibility of Hakka culture brand image design by means of a questionnaire survey, in which 873 questionnaires were distributed and 579 questionnaires were recovered. The survey results show that mobile phone cases are the most popular in this Hakka culture brand image design, with a popularity of 97%.

## Hakka culture brand image design

**FIGURE 1:**
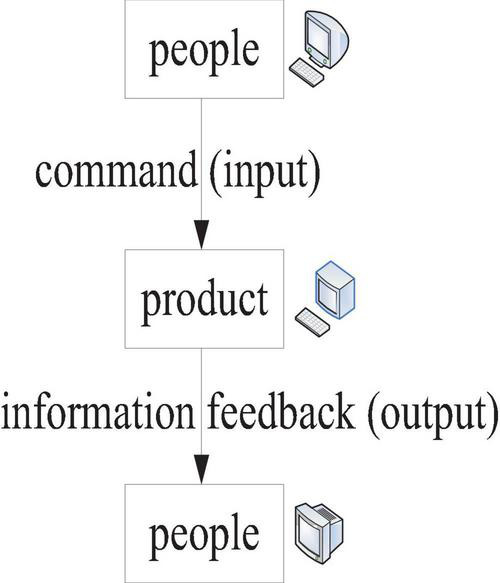
Human–computer interaction process.

The process of human–computer interaction is shown in Figure 1.

**FIGURE 2:**
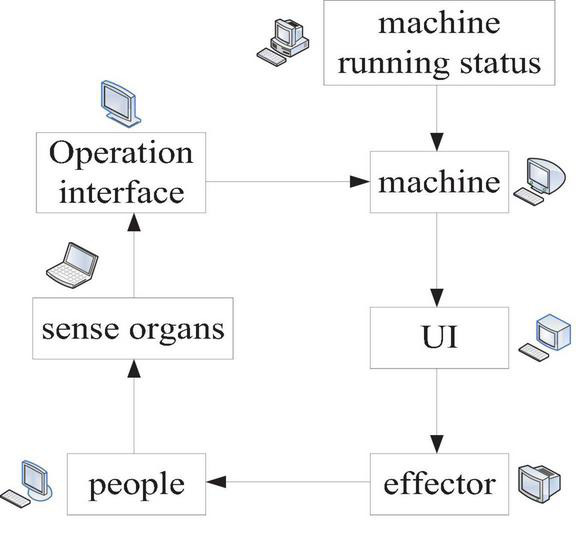
Traditional human–machine interface interaction.

The traditional human–machine interface interaction is shown in Figure 2.

The human–computer interaction in this article is mainly reflected in the gesture recognition of elements in brand design. Color modeling in branding design is based on Bayesian, if a point in the image belongs to the color class, then:


(1) $$𝔄\left(𝔅|ℭ\right)=𝔄\left(\bar{𝔅}|ℭ\right)$$

𝔅 indicates that the pixel belongs to the color class and $\bar{𝔅}$ does not.

According to Bayesian theory:


(2) $$𝔄\,\left(𝔅|ℭ\right)=\frac{𝔄\,\left(ℭ|𝔅\right)\,𝔄\,\left(𝔅\right)}{𝔄\,\left(ℭ\right)}$$

So


(3) $$\frac{𝔄\,\left(ℭ|𝔅\right)\,𝔄\,\left(𝔅\right)}{𝔄\,\left(ℭ\right)}=0.5$$

It has


(4) ℭ⁢ϵ⁢𝔅

(5) $$𝔄\,\left(ℭ|𝔅\right)=\frac{{𝔇}_{𝔅}\,\left(ℭ\right)}{{𝔈}_{𝔅}}$$

𝔈_𝔅_ is the total number of color points and 𝔇_𝔅_(ℭ) is the histogram obtained by counting color samples.


(6) $$𝔄\,\left(ℭ\right)=\frac{{𝔇}_{𝔉}\,\left(ℭ\right)}{{𝔈}_{𝔉}}$$

(7) $$𝔄\,\left(𝔅\right)=\frac{{𝔈}_{𝔊}}{{𝔈}_{𝔉}}$$

𝔈_𝔉_ is the total pixel points, and 𝔈_𝔊_ is the total number of sample color points.


(8) $$\frac{{𝔇}_{𝔅}\,\left(ℭ\right)}{{𝔇}_{𝔉}\,\left(ℭ\right)}=\frac{{𝔈}_{𝔅}}{2\,{𝔈}_{𝔊}}$$

Detection of color points:


(9) $$𝔍\,\left(𝔏,𝔐\right)=\left\{ \begin{array}{cc} 255 & 77=\left(𝔏,𝔐\right)=127\\ 0 & \text{otherwise} \end{array}\right.$$

**FIGURE 3:**
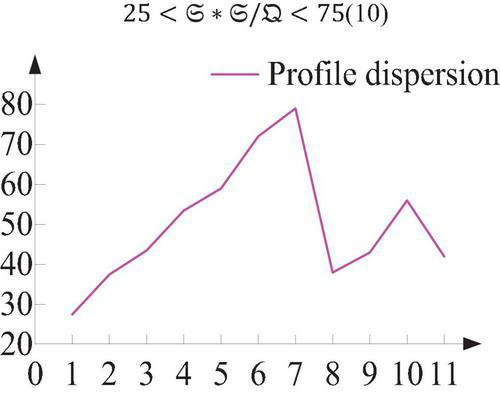
Gesture profile dispersion.

Let the area of the outline of the color block be 𝔔, the length of the outline be 𝔖, and the dispersion of the outline of the gesture has nothing to do with the size of the gesture, as shown in Figure 3.


(10) 25=𝔖*𝔖/𝔔=75

The problem of optimal fitting with an ellipse is transformed into:


(11) $$\text{min}\,{||𝔘\,𝔛||}^{2},s.t.{𝔛}^{\text{T}}\,𝔜\,𝔛=1$$

(12) $$2\,{𝔘}^{\text{T}}\,𝔘\,𝔛-2\,ℨ\,𝔜\,𝔛=0,s.t.{𝔛}^{\text{T}}\,𝔜\,𝔛=1$$

ℨ is the Lagrangian factor.

Initial target model:


(13) $$\text{a}\,\left(𝔏\right)=\sqrt{{\left(𝔏-{𝔏}_{g}\right)}^{\text{T}}\,\left(𝔏-{𝔏}_{g}\right)}$$

Probability of the target point:


(14) $$o\,\left(𝔏\right)=\text{log}\,\left(\frac{\kappa \,\left(𝔐|\delta_{0}\right)}{\kappa \,\left(𝔐|\delta_{1}\right)}+1\right)$$

δ_0_ and δ_1_ are the color models of the background and target, respectively.


(15) $$\kappa \,\left(𝔐\right)=\sum_{\mu }^{-1}\left(𝔐-\frac{\delta_{0}}{\delta_{1}}\right)$$

**FIGURE 4:**
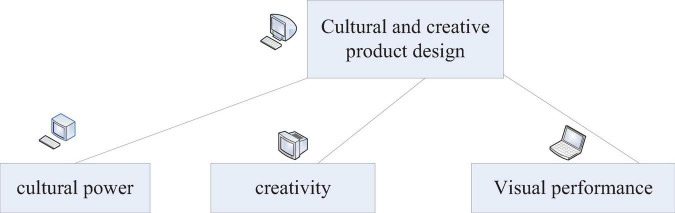
Criteria for cultural and creative product design.

Brand cultural and creative products are the abbreviations of brand cultural and creative products. The most important connotation and essence of brand cultural and creative products are their unique cultural characteristics and innovative ideas. Through a specific form carrier, it is conveyed to the public in the form of visual images (Sappington, 2017; Yoon et al., 2017). Literally speaking, the standards of cultural and creative product design (Figure 4) can be divided into three parts: cultural power, creativity, and visual performance (Lee et al., 2017b; Ratcliffe, 2021).

The brand cultural and creative products of the new cultural industry formed by the integration of the cultural industry and the creative industry must have cultural and creative elements (Anna et al., 2019; Lee et al., 2021). Moreover, branded cultural and creative products are products obtained by integrating culture and creativity. Its unique cultural connotation and innovative design concepts must be conveyed through a visual image that the public can interpret, so its visual components are also extremely important. Therefore, when a designer creates and designs brand cultural and creative products, he should comprehensively study the cultural, creative, and visual elements of his cultural and creative products (Bean, 2017; Murray et al., 2017).

The cultural elements of brand cultural and creative products are the inheritance and development of excellent traditional cultural genes (Jonathan, 2017; Kim and Lee, 2019). Through the use of modern artistic techniques and new art forms, it has created a new brand of cultural and creative products that meet the spirit and aesthetic needs of modern people (Paul, 2018; Lee and Park, 2019). In the specific design practice, it is necessary to dig deep into the excellent cultural elements. It makes it as much as possible to extract a modeling language that can reflect its cultural characteristics and pays attention to the utility of extraction. At the same time, when integrating traditional cultural elements, it must have high aesthetic value. At the same time, it is necessary to prevent the excessive pursuit of cultural expression and affect its use function (Zeitune, 2021).

The elements of creativity require brand cultural and creative products to give people a “reasonable, unexpected” design psychological feeling through their visual performance. The creativity should not only reflect surprise and novelty but also exquisiteness and ingenuity. In terms of creative expression, it should be intuitive, concise and visually impactful, and easy to identify and interpret. However, sometimes when expressing complex and tactful or rich visual effects, it is easy to be monotonous, and more elements can be creatively combined to better reflect the richness of visual effects (Ibrahim et al., 2020).

The visual elements are the visual display of the culture and creativity of the brand cultural and creative products. Generally speaking, it is the superficial visual experience of the brand cultural and creative products, which is composed of the text, graphics, and three elements of the brand cultural and creative products (Takamura and Suzuki, 2018). Words are an important means and the most direct way for visual symbols to transmit relevant information and cultural connotations. Designers should carefully design and arrange these texts carefully, because the quality of text design directly affects the expression of its meaning and affects the quality of brand cultural and creative products. Good text design can not only give people a sense of wonder, give people a visual enjoyment, but also can convey the meaning to be conveyed. Graphics are one of the factors of visual composition, and it has a strong appeal and a more intuitive expression effect. It can truly, effectively, and accurately make the audience feel the cultural connotation, and then make the audience of the brand cultural and creative products more acceptable. When the graphic elements express the cultural connotation to be expressed, they must not only be clear, accurate, and true. It is an important part of the visual elements of brand cultural and creative products, which can make the audience better recognize and accept the cultural connotation brought by it, and it has a certain degree of creativity. From the perspective of the color components of visual components, after related investigations, it is found that when viewing objects, the initial color perception accounts for a large proportion. And the shape is only a small part. Therefore, we can see that in cultural creativity, color is a very important factor, which can make consumers have a first impression of it, thus affecting consumers’ choices. Therefore, in the visual design of brand cultural products, color matching is essential. The colors of Hakka clothing are shown in Table 1.

**TABLE 1:** Hakka clothing colors.

Numbering	Color	Numbering	Color
1	White	4	Blue
2	black	5	Blue
3	gray	6	Red

First, to sum up, when designing and developing tourism brand cultural and creative products, it is necessary to excavate the excellent cultural connotation of the location and use abstract or figurative extraction methods to transform them into cultural elements of brand cultural and creative products. Second, it organically integrates cultural elements and creative elements and loads its products through visual elements, so as to present a high-quality brand cultural and creative product to the tourist consumers.

As a tourism brand cultural and creative product in the two disciplines of cross-culture and tourism, its design and development must have cultural connotation and cultural elements, so as to play the function of carrying and disseminating the essence of Gannan Hakka culture. It has to be innovative, it should have creative components, and it can stimulate the curiosity of consumers. Based on this, the integration of Hakka culture into the cultural and creative design of tourism brands should start with the extraction and design methods of element symbols.

**FIGURE 5:**
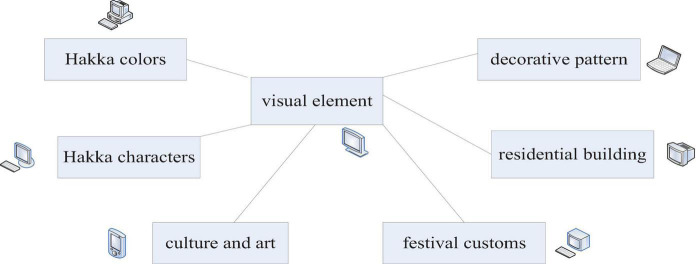
Visual elements of Hakka culture brand image.

First of all, the extraction of Hakka cultural elements should not be too abstract, and its original and intuitive characteristics should be retained. It enables tourism consumers to fully perceive the cultural charm and spiritual enjoyment brought by Hakka culture. Second, cultural and creative products belong to the discipline of product design. The cultural and creative design of Hakka cultural tourism brands should not be too rigid. But from the perspective of modern aesthetics, it finds the point of integration with tourism brand cultural and creative products through the refinement of Hakka cultural connotation and realizes the visual transformation of cultural connotation. The visual symbol of the Hakka culture brand image is shown in Figure 5.

For the design of cultural and creative products of tourism brands, it is necessary to present the connotation of Hakka culture in a creative form. It mainly builds the brand’s visual system through elements such as pattern symbols, decorative colors, and text styles. And the construction of a unified visual system is the key to the success of the design and construction of cultural and creative products of tourism brands.

Tourism brand cultural and creative products are based on the combination of culture and products under the construction of tourism brands. Its core is to use a series of cultural and creative products to spread regional culture through the construction of tourism brands. In recent years, China’s tourism industry has shown a trend of steady growth. As a result, it also ushered in an upsurge of people’s love for cultural and creative products. Therefore, through the establishment of the Hakka cultural tourism brand, a series of cultural and creative product designs are designed. It is an important way to inherit and promote Hakka culture.

In the era of the Internet, the way for people to obtain information has provided convenience, and at the same time, it has also caused an impact on the excellent local culture, which has further intensified the market competition of local tourism cultural and creative products. Therefore, it is an important opportunity to inherit and carry forward the Hakka culture by using the tourism brand cultural and creative product design. By creating tourism cultural and creative products full of Hakka cultural connotations, it can deconstruct the value of Hakka culture.

Redesigning and innovating Hakka culture through tourism brand cultural and creative products can make tourism cultural products possess spiritual culture and integrate into modern life, thereby creating value. At the same time, in the design process of cultural and creative products of tourism brands, it is necessary to continuously absorb excellent Hakka cultural genes and excellent Hakka spirit, and then create value through redesign and innovation. In addition, the parts of tourism cultural and creative products that are difficult to interpret in Hakka culture should be simplified. It in turn makes it a form or product that the public can easily accept, which enables more people to deeply understand Hakka culture, thereby creating value and inheriting Hakka culture.

The research on the visual image design of cultural and creative products of Gannan tourism brands requires us to continue to explore and try. It involves the living customs, architectural features, patterns, symbols, and color expressions of the Hakka in Gannan, all of which require in-depth research. Only in this way can we design a cultural and creative visual image of tourism brands that the public loves and has cultural value.

**FIGURE 6:**
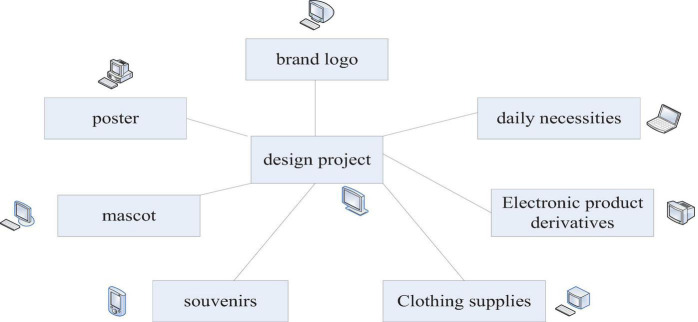
Hakka culture brand image design project.

Here, only preliminary design research is carried out on the brand logo, poster promotion, and mascot image promotion of the cultural and creative products of the Hakka tourism brand in Gannan, and it is hoped that this method can be passed. It allows the public to better understand the Hakka culture in Gannan, and let the Hakka culture in Gannan enter the hearts of the people. And innovation is carried out on the basis of inheritance, which enriches the connotation of Hakka cultural resources in Gannan. By focusing on creating the core brand cultural and creative products of Gannan Hakka tourism, it truly shapes the visual image of the cultural and creative products of the southern Gannan Hakka tourism brand. The Hakka culture brand image design project of this article is shown in Figure 6.

The logo is an important language symbol in the communication of information, which symbolizes the corporate image and culture. An excellent enterprise has an important symbol to convey the corporate image and culture, such as Tencent’s penguin and Huawei’s petals, the logos of these companies penetrate deeply into the psychology of consumers, making people connect with the concept that the company wants to convey at the first time. An excellent tourism brand image logo can help consumers better understand the concept to be conveyed, which requires designers to be concise, not complicated, and highly recognizable in the design stage.

The Gannan area is a city with rich and unique Hakka culture, with profound historical accumulation. The “Hakka enclosure” in Gannan is the epitome of Hakka culture, so this article mainly takes the Hakka enclosure as the main design element. The abstract forms combine to form the upper half of the logo graphic. And the abstract form of the wall of the Hakka enclosure is combined with the abstract form of the initial letter “N” of the word “South” to form the lower half of the logo graphic. “Gannan Hakka” in the logo uses the simplified version of the Founder’s Book, while “GANNAN KEJIA TRAVEL” uses the simplified version of Zhang Haisheng’s sharp line. Finally, it is decorated with blue of #004ccf and red of #973f37. And it uses standardized mapping. As a result, the logo of the Gannan Hakka tourism brand was designed.

The use of posters to convey information is ubiquitous in our lives, and it exists in the perceptual field of various images with visual impact. In the early days of word dissemination, words, as the carrier of information dissemination, are a very important form of expression, which can be used to transmit information. With the rapid development of society and information technology, media such as television, computers, and websites have appeared. These communication media use images as the main form of presentation, so the visual carrier that uses images as the main information disseminates its importance. In today’s fast-paced life, it takes a certain amount of time to accept textual information, but it is clear at a glance to accept the information conveyed by images. Humans have entered the era of conveying information through images. This means that when designing the posters of the Gannan Hakka tourism brand, we should pay attention to the way of design and processing. In addition to paying attention to the aesthetics of the design, we must also ensure the accuracy of information transmission and ensure that the information can be conveyed directly and quickly. The design of the promotion poster is to take the extracted visual elements such as the Hakka characters in Gannan, the festival lion dance, and the Hakka enclosure in Gannan as the main visual elements. With the images with Gannan characteristics as the background, the colors are mainly red and blue, and the entire visual experience is in the form of paper cuts. The shape of the paper cut is the image of the Hakka enclosure, so as to create and design posters with the characteristics of the Hakka culture in Gannan. It allows tourism consumers to experience the Hakka culture in Gannan more intuitively.

The Hakka culture in Gannan inherits the Confucian and Taoist culture and auspicious culture in the traditional Han culture. Therefore, it is of great value and significance to use the Confucian and Taoist culture and the auspicious culture as the content of the promotion posters for the Hakka tourism brand in Gannan. For the design of such posters, this article first extracts the visual symbols of the Hakka enclosures in Gannan and the Hakka ancestral hall in Gannan and designs them to have the word “Gannan” with Hakka charm. Second, it is combined with Hakka architectural decoration, Hakka square enclosure, and Hakka lion dance, and uses “circle” as an auxiliary design element, and finally adds the words of auspicious culture, Taoism culture and Confucian culture. From this, a visual image with auspicious meanings is designed that can reflect the “round sky and place” of Taoism and the “respect of ancestors” of Confucianism.

The Hakka cultural connotation and decorative beauty contained in the decorative patterns of Gannan Hakka are irreplaceable. In order to better publicize the Hakka culture and better allow tourists to understand the Hakka culture in Gannan, this article will contain the visual symbols that enrich the Hakka culture and make a series of posters for publicity and promotion design. The theme words of the posters of the series of Hakka decorative pattern posters are the image of “Gannan Hakka” presented in the form of seals, and the “Gannan Hakka” using the font of Founder Bangshu. Its main design elements are the combined layout of the Hakka visual symbols in Gannan extracted in this article, and the colors also use the extracted red and blue. The whole visual effect is to present a dynamic process of turning pages, symbolizing the rich cultural value of the decorative patterns of Gannan culture.

The Hakka enclosure buildings in Gannan, the tea-picking drama, and the unique charm of women in the Hakka customs are the embodiment of the essence of the Hakka culture in Gannan. Therefore, it is also essential to use it as the content of poster promotion. For this type of poster promotion design, the theme words are mainly the graphic design of fonts and related elements for the content of “enclosure,” “tea-picking opera,” “lion dance,” and “custom.” Its “enclosed house” is combined with green tiles, “tea-picking opera” is combined with tea leaves, and “lion dance” is combined with elements of lion dance. In addition, in the Hakka life customs, whether it is sacrifices or festivals, it is the Hakka women who are preparing the food they need, so the “custom” is combined with the food. In terms of graphic elements, the materials such as “enclosed house” and “tea garden” are contrasted in gray and color with the extracted visual elements such as the enclosed house, tea-picking opera, lion dance, and Hakka women. This made a series of poster promotion designs.

Under the circumstance that enterprises inherit the traditional Chinese auspicious art and the modern business competition is fierce, the brand mascot is a specific image created in order to obtain the consumer psychology that consumers seek auspiciousness and establish a brand image. The auspicious meaning contained in the brand mascot coincides with the auspicious meaning in Hakka culture. Therefore, it is essential to design and create the mascot of the Gannan tourism brand to promote the Hakka culture of Gannan. Regarding the mascot design of the Gannan tourism brand, its main image is to use the image of children. Children represent innocence and simplicity, which represents the simple life attitude of the Hakka people. The image of the child wearing a tiger hat and a dragon on the chest represents the Hakka people in Gannan seeking profit and warding off evil spirits and yearning for a better life. In the color ratio of the entire mascot, the main red and blue colors in Hakka are also used. And this article will serialize the design.

Each ethnic group or region has its own unique history and culture. With the development of tourism, cultural and creative products of tourism brands are also popular among tourists. The following series of cultural and creative tourism brand cultural and creative products mainly carry out a series of creative designs for the Hakka culture in Gannan. There are not only commemorative souvenirs and daily necessities full of life flavor but also humane clothing items and electronic product derivatives with scientific and technological charm. After designing the cultural and creative products of the Gannan Hakka tourism brand, it is believed that in future, using this method to spread and carry forward the Gannan Hakka culture and even the excellent traditional culture of various parts of China has a good application prospect.

This article will extract the Hakka cultural symbols from the design of cultural and creative products of the Gannan tourism brand. It is used in souvenirs, clothing items, daily necessities, and derivatives of electronic products. By combining the visual patterns and tones of the cultural and creative products of the Gannan Hakka tourism brand, it uses tourism as a means of communication to convey the Hakka cultural spirit and aesthetic value. It will carry forward the excellent traditional culture.

As the tourism souvenir market in Gannan has begun to take shape, its tourism consumption market in Gannan has shown a growing trend. Therefore, in order to better carry forward and inherit the Hakka culture of Gannan, the design of cultural and creative products of the Hakka cultural tourism brand in Gannan has introduced the design of tourist souvenirs.

With the passage of time, the badge has gradually been given more meanings, its scope of application has also extended to all aspects, and it has also given specific commemorative meanings in tourism brand products. Therefore, the design and development of badges as souvenirs for the Hakka cultural tourism brand in Gannan is also extremely important. In the design of badges and souvenirs for the Hakka cultural tourism brand in Gannan, the main colors are also the blue of #004ccf and the red of #da5038. The main visual image is extracted from the Hakka women as the main visual image, and combined with the traditional patterns of clouds, so as to design badge commemorative products that represent regional characteristics and traditional meanings.

Stamps are the most collected souvenirs and collectibles today, and are known as “miniature works of art.” It is essential to use stamps as one of the souvenirs of the cultural and creative brands of Gannan Hakka tourism. The stamp design of the Gannan Hakka cultural tourism brand is loaded with the unique blue and red of the Gannan Hakka, and it reorganizes the visual elements such as the refined Gannan Hakka lion dance and Gannan Hakka women with traditional auspicious patterns, so as to design the hard-working spirit of Hakka women. It has commemorative stamps that convey the auspicious meaning of seeking profit and avoiding evil.

Bookmarks originated from the Warring States Period and were called “toothpicks” at that time to mark the progress of reading. The continuation to this day not only maintains its function but also increases the commemorative significance of the gift. The main design method of bookmark design of the Hakka cultural tourism brand in Gannan is to hollow out the decorative patterns of Hakka in Gannan extracted in this article. This design method not only conveys the carving beauty of Hakka architectural decoration in Gannan but also reflects the cultural meaning contained in the decorative patterns.

Cultural calendar is to add cultural color on the basis of the traditional calendar. The external form of the calendar is injected into its novelty and interest, so that consumers can feel the charm of cultural connotation in the daily life of using the calendar. This is also the promotion and dissemination of traditional culture. The visual image design of the cultural calendar of the Gannan Hakka tourism cultural and creative brand is not only practical and cultural but also has tourism commemorative significance. The visual image design of the cultural calendar of the Gannan Hakka tourism cultural and creative brand is mainly based on the red color number #da5038 extracted in this article as the main color effect. The main visual image is the extracted festival customs symbols, and the abstract “mountain,” “swallow,” and “cloud” are used as a foil to create an atmosphere of harmonious coexistence with nature.

Clothing items can often reflect people’s living customs and emotional needs. Moreover, clothing is a kind of cultural and artistic manifestation, through which it can convey the cultural and aesthetic tendencies of a specific region and historical period. The artistry of clothing is expressed and reflected through certain visual elements of clothing. Therefore, it is also essential to apply the extracted visual symbols of Gannan Hakka to the cultural and creative products of Gannan Hakka tourism brands. In this article, the design and development of tourism clothing and accessories for the cultural and creative products of the Hakka tourism brand in Gannan include silk scarves, tourist hats, and t-shirts. As for the color presentation of these clothing items, in terms of color use, the blue of #004ccf and the red of #da5038 are mainly combined. At the visual pattern level, first of all, silk scarves are women’s favorite clothing accessories. The visual pattern design of silk scarves is to repeat the design of the extracted visual symbols of the Hakka decorative patterns in Gannan and use red and blue colors alternately to design a visual pattern of silk scarves that can reflect the charm of Hakka culture.

Second, the tourist hat is a very popular cultural and creative product in the tourism market. This article designed the tourist hat in the cultural and creative products of the Hakka tourism brand in Gannan. They are reorganized with the extracted Hakka women and the visual symbols of Gannan Hakka, and then use the traditional clouds and mountains as auxiliary design elements. It also applies the blue of #004ccf and the red of #da5038 to travel caps. The visual patterns of two different tourist hats are designed, which not only have visual aesthetics but also fully reflect the spiritual charm of Gannan Hakka.

Finally, t-shirts are commonly used clothing in people’s daily life. The pattern design of the t-shirt is to combine the extracted festival customs of Gannan with the visual symbols of the Hakka tea-picking opera in Gannan and traditional water patterns. It designs different t-shirt visual patterns and uses them in t-shirts.

Among the tourism cultural and creative products, their daily necessities are often paid attention to and purchased. During our travel, we often see some cultural and creative daily necessities with rich cultural heritage and aesthetic sense, and we will buy them at home and use them in our daily life. Therefore, as a daily necessity, it has a high function of spreading culture. This article regards cups, canvas bags, and pillows as the cultural and creative daily products of the Gannan Hakka tourism cultural and creative brand. In the use of color elements in the design, the extracted blue of #004ccf is combined with the red of #da5038. In terms of the main visual image, the design of the cup is mainly to extract the visual symbols of the lion dance of the Gannan Hakka and the tea-picking opera of the southern Gannan Hakka. It forms visual patterns with traditional elements such as “mountain,” “water,”, “pavilion,” and “cloud,” showing the charm of traditional landscape painting. And it designs a visual pattern of a cup based on red tones and designs a visual pattern based on blue tones. In addition, decorating it in the cup can not only reflect the spiritual connotation of Gannan Hakka culture but also show the beauty of traditional landscape painting.

The visual pattern design of the canvas bag is similar to the visual pattern design of the cup. The visual pattern of a cup is also designed mainly in red, and a visual pattern is designed in blue. The red color is the combination of southern Gannan Hakka women and traditional elements such as “mountain,” “cloud,” and “pavilion” to design a tranquil and simple design pattern. The blue color is mainly used to draw the abstract nature of the ocean. The ocean represents life and has the meaning of endless life, which is consistent with the spiritual pursuit of the Hakka people in Gannan. And combined with the visual symbols of the tea-picking opera in Gannan extracted in this article, it can not only reflect the endless life view of the Hakka in Gannan but also reflect its hard-working spirit. Finally, the visual pattern of the design is applied to the canvas bag to convey the cultural spirit it carries to the public.

The design of the pillow is to arrange and combine the visual symbols of the decorative patterns of the Hakka in Gannan, which has a strong cultural implication, and use different background colors to design the design of the pillow. The background color of different brightness will cause different visual experiences, so when it is applied to the pillow, it will also have different visual effects.

Since ancient times, there has been a tradition of women dressing up by mirrors, and until now, every woman carries a mirror with her. As a result, mirrors have become one of the must-have items in today’s women’s lives. The mirror of the cultural and creative products of the Hakka tourism brand in Gannan, this article has made the main design in its visual image. By using the blue of Gannan extracted in this article as the main color, it also combines the extracted Hakka enclosure with the abstract mountain to design the mirror design of the Gannan tourism brand with Hakka charm in Gannan.

With the development of technology, electronic products are loved by young people. The enthusiasm for matching derivative products has also risen sharply. Therefore, it is also extremely important to regard the derivatives of electronic products as cultural and creative products of the Hakka tourism brand in Gannan. This article focuses on the design of mobile phone cases and headphones in electronic product derivatives. In many tourist consumer groups, they will buy earphone products that can produce emotional memories of tourist destinations. In the design of the electronic product derivatives of the Gannan Hakka tourism brand, the color elements of the headphones mainly use the blue of #004ccf and the red of #da5038. The graphic elements mainly use the extracted visual symbols of the Hakka living customs and Hakka architecture in Gannan and decorate the appearance of the earphones, respectively, to design earphones of different styles.

Mobile phone cases are not only to meet people’s daily needs of protecting mobile phones but also to the needs for emotional sustenance. When people travel, they often have the desire to buy mobile phone protective cases that can reflect local cultural emotions and aesthetics. Therefore, it is very important to use the mobile phone protective case as one of the electronic derivatives of the Gannan Hakka tourism brand. In the visual pattern design of the mobile phone protective case, the main color used is also the extracted blue of #004ccf and red of #da5038 as the main color elements of the design. Its image elements are also the visual design of various styles of elements such as the southern Gannan Hakka tea-picking opera, the southern Gannan Hakka lion dance, Gannan Hakka women. The visual symbols of Gannan Hakka architecture, and the abstract “mountain,” and the “cloud” extracted in this article. And, it will extract these visual patterns and use them in the phone case. It can not only resonate emotionally with the people but also inherit traditional culture.

To sum up, it can be seen that the products of this Hakka culture brand image design are shown in Tables 2, 3.

**TABLE 2:** Product 1 of Hakka culture brand image design.

Numbering	Product	Numbering	Product
1	Logo	4	Badge
2	Poster	5	Stamp
3	Mascot	6	Bookmark

**TABLE 3:** Product 2 of Hakka culture brand image design.

Numbering	Product	Numbering	Product
7	Cultural calendar	11	Throw pillow
8	T-shirt	12	Mirror
9	Cup	13	Phone case
10	Canvas bag	14	Earphone

## Brand visual image

This research is to verify the feasibility of the Hakka cultural brand image design through a questionnaire survey. Eight hundred seventy-three questionnaires were distributed and 579 questionnaires were recovered. The results of the survey on the way tourists understand cultural products are shown in Table 4.

**TABLE 4:** Understanding pathway survey results.

Way	Proportion	Way	Proportion
Recommended by acquaintances	31.7%	Travel agency	14.3%
National festivals	25.3%	Magazines	3.40%
Internet platform	24.2%	Other ways	1.10%

Table 4 shows that the proportion of tourists who understand the brand image of Hakka culture through the recommendation of acquaintances is relatively high, accounting for 31.7%, followed by ethnic festivals, accounting for 25.3%.

**FIGURE 7:**
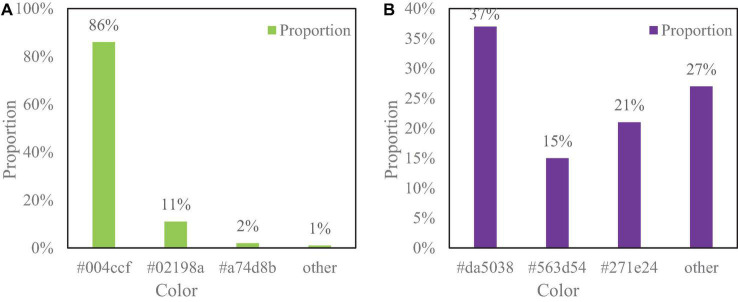
The proportion of different clothing colors. **(A)** Shows that blue with the color number #004ccf has the largest proportion. **(B)** Shows that the red with the color number #da5038 has the largest proportion, followed by the purple color of #563d54 and the color number #271e24 also occupy a certain proportion. The main colors extracted from this are #004ccf, #973f37, #563d54, and #271e24.

There are two kinds of Hakka costumes designed in this article, one of which has the color composition of #004ccf, #02198a, #a74d8b, and others, and the other is the color composition of #da5038, #563d54, #271e24, and others. The specific color ratio is shown in Figure 7.

Figure 7A shows that blue with the color number #004ccf has the largest proportion. Figure 7B shows that red with the color number #da5038 has the largest proportion, followed by the purple color of #563d54 and the color number #271e24 also occupy a certain proportion. The main colors extracted from this are #004ccf, #973f37, #563d54, and #271e24.

The preferences and expectations of tourists for which original cultural images to promote are shown in Table 5.

**TABLE 5:** Preferences and expectations.

Primitive culture	Proportion	Primitive culture	Proportion
historical buildings	18%	Craftsmanship	14%
Historical and cultural landscape	13%	Folk belief	9%
Natural environment resources	8%	Folklore	8%
Representative material culture	15%	Performance culture	15%

Table 5 shows that the promotion of historical buildings is the highest among tourists, accounting for 18%, followed by representative material culture, accounting for 15%.

**FIGURE 8:**
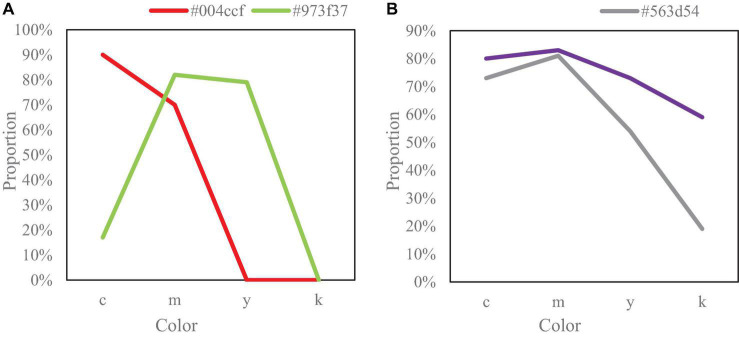
CMYK of primary colors drawn. **(A)** Shows CMYK of #004ccf with 90% cyan, 70% magenta (magenta), and zero for the other two colors. **(B)** Shows CMYK of #563d54 with 73% cyan, 81% magenta (magenta), 54% yellow, and 19% black.

The extracted CMYK (cyan, magenta, yellow, and black) of the main colors are shown in Figure 8.

Figure 8A shows CMYK of #004ccf with 90% cyan, 70% magenta (magenta), and zero for the other two colors. Figure 8B shows CMYK of #563d54 with 73% cyan, 81% magenta (magenta), 54% yellow, and 19% black. The CMYK of #271e24 is 80% cyan, 83% magenta (magenta), 73% yellow, and 59% black.

**FIGURE 9:**
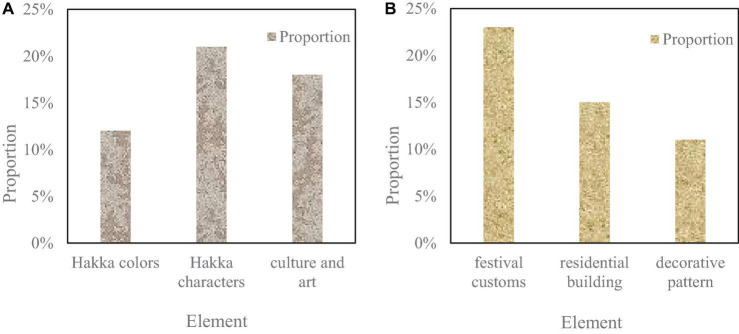
Distribution of visual symbols in Hakka cultural brand image. **(A)** Shows that the proportion of Hakka colors is 12%, and the proportion of Hakka characters is 21%. **(B)** Shows that the proportion of festival customs is 23%, the proportion of residential buildings is 15%, and the proportion of decorative patterns is 11%. The proportion of solar terms and customs visual symbols is the highest, indicating that this design attaches great importance to solar terms and customs. In addition, although the proportions of different visual symbols are different, they are not very different, indicating that this design has considered different visual symbols of Hakka culture, which also expresses the comprehensiveness of this design.

The distribution of visual symbols in the Hakka cultural brand image is shown in Figure 9.

Figures 9A,B show that the solar terms and customs visualsymbols occupy the highest proportion, indicating that this design attaches great importance to the solar terms and customs. In addition, although the proportions of different visual symbols are different, they are not very different. It shows that this design has considered different visual symbols of Hakka culture, which also expresses the comprehensiveness of this design.

**FIGURE 10:**
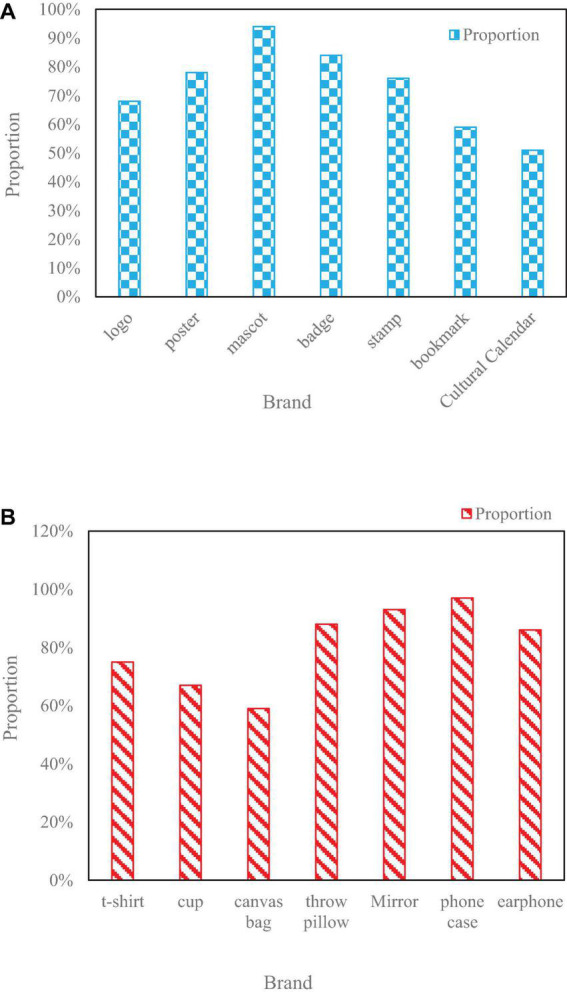
Popularity of brands in different cultures. **(A)** Shows that the logo accounts for 68% of the Hakka culture brand image design, 78% for the poster, and 94% for the mascot. **(B)** Shows that the mobile phone case is the most popular in this Hakka culture brand image design, and the popularity is as high as 97%. At the same time, the popularity of different products is above 50%, which also indirectly shows that the design of this design effectiveness.

The popularity of different cultural brands is shown in Figure 10.

Figures 10A,B show that the mobile phone case is the most popular in this Hakka culture brand image design, with a popularity of 97%, and the popularity of different products is above 50%. This also indirectly shows the effectiveness of this design.

## Conclusion

In order to increase the interactive experience between tourists and brand culture, this article designs the brand image of Hakka culture through the human–computer interaction model. Based on the analysis and arrangement of Hakka culture in Hakka culture articles, this article proposes the manifestations of Gannan Hakka culture. It includes visual symbols of Gannan Hakka culture color, visual symbols of visual characters, visual symbols of culture and art, visual symbols of festival customs, visual symbols of residential buildings and visual symbols of decorative patterns. And it was applied to the visual image design of the Hakka cultural tourism brand in Gannan. In this plan, the visual image design of the Gannan Hakka cultural tourism brand and the series of cultural and creative products of the Gannan Hakka cultural tourism brand are designed. Visual image promotion design mainly includes in-depth design of trademark and affiliated patterns, poster publicity and promotion design, and image promotion design. The Hakka culture brand image designed this time is not rich enough, and it can be expanded in the following research. It is hoped that the design and development of the Hakka tourism brand in Gannan will not only promote the development of tourism in Gannan but also promote the development of the tourism industry, and promote the inheritance of Hakka culture in Gannan and the inheritance of traditional Chinese culture.

## Author’s note

RC was born in Ganzhou, Jiangxi, China. In 2004, she received a bachelor’s degree from Shaanxi University of Science & Technology, China, and a master degree in 2007. Now, she works in the College of Arts and Design, Gannan Normal University. Her research interests include graphic design, brand design, and user interface design. E-mail: chenrui@gnnu.edu.cn.

## Data availability statement

The original contributions presented in the study are included in the article/supplementary material, further inquiries can be directed to the corresponding author.

## Ethics statement

Ethical review and approval was not required for the study on human participants in accordance with the local legislation and institutional requirements. Written informed consent from the (patients/participants OR patients/participants legal guardian/next of kin) was not required to participate in this study in accordance with the national legislation and the institutional requirements.

## Author contributions

RC: conceptualization, methodology, and writing.
